# Cortical processes of multisensory plausibility modulation of vibrotactile perception in virtual environments in middled-aged and older adults

**DOI:** 10.1038/s41598-024-64054-z

**Published:** 2024-06-11

**Authors:** Kathleen Y. L. Kang, Robert Rosenkranz, Mehmet Ercan Altinsoy, Shu-Chen Li

**Affiliations:** 1 Centre for Tactile Internet with Human-in-the-Loop (CeTI), Technische Universität Dresden, Dresden, Germany; 2 Faculty of Psychology, Technische Universität Dresden, Zellerscher Weg 17 Room A232/233, 01069 Dresden, Germany; 3 School of Psychology and Vision Sciences, University of Leicester, Leicester, UK; 4 Faculty of Electrical and Computer Engineering, Technische Universität Dresden, Dresden, Germany

**Keywords:** Aging, Multisensory processing, Tactile perception, Contextual expectation, Virtual reality, fNIRS, Neuroscience, Psychology

## Abstract

Digital technologies, such as virtual or augmented reality, can potentially support neurocognitive functions of the aging populations worldwide and complement existing intervention methods. However, aging-related declines in the frontal-parietal network and dopaminergic modulation which progress gradually across the later periods of the adult lifespan may affect the processing of multisensory congruence and expectancy based contextual plausibility. We assessed hemodynamic brain responses while middle-aged and old adults experienced car-riding virtual-reality scenarios where the plausibility of vibrotactile stimulations was manipulated by delivering stimulus intensities that were either congruent or incongruent with the digitalized audio-visual contexts of the respective scenarios. Relative to previous findings observed in young adults, although highly plausible vibrotactile stimulations confirming with contextual expectations also elicited higher brain hemodynamic responses in middle-aged and old adults, this effect was limited to virtual scenarios with extreme expectancy violations. Moreover, individual differences in plausibility-related frontal activity did not correlate with plausibility violation costs in the sensorimotor cortex, indicating less systematic frontal context-based sensory filtering in older ages. These findings have practical implications for advancing digital technologies to support aging societies.

## Introduction

Older populations are increasing rapidly worldwide, yielding important economic, social, and individual implications. Recent estimates by the World Health Organization^1^ indicate that on average more than 15% of the populations worldwide will be 65 years or older by 2030. There are also substantial variations in the extent of population aging around the globe and in some regions the estimates are even higher (e.g., 22% in Europe and North America; 19.4% in Australia and New Zealand; 16.3% Eastern and South-Eastern Asia). Aging attenuates the functions of various brain networks at the neuroanatomical^2^, neurochemical^3^, and neurofunctional^4,5^ levels. These aspects of brain aging underlie various cognitive^6^ and perceptual^7,8^ impairments in old ages. These declines tend to progress gradually during later periods of the adult lifespan and have their onsets already in middle age^6–8^. Facing challenges of global aging, it is crucial to leverage new technologies to buffer declining neurocognitive functions in older adults and to complement existing intervention methods^9^. To this end, it is important to better understand how neurocognitive declines in older ages may require special adjustments of the envisioned technological supports to develop age-inclusive technologies^10^ to serve the growing aging populations.

Advances in communication and other digital technologies in the past decade are creating new digital environments for humans^10^. Of note, virtual or augmented reality (VR/AR) are increasingly used in lab-based experiments to enable more naturalistic studies of human behaviours^11,12^. Their applications in older populations^13,14^ are also currently being explored. However, there are still considerable concerns about the discrepancies between real and virtual experiences^15^, which are due, in part, to the main reliance on vision and hearing without integrating other modalities^16^. Furthermore, for gerontological applications it would be necessary to evaluate age-related differences in neurocognitive processes of multisensory perception and action enroute to the developments of suitable VR-/AR-technologies to exploit them for augmenting perceptual and cognitive functions in old age^10^.

The sense of touch operates with a more precise temporal resolution than vision and hearing^17^; therefore, including tactile information into digitalized environments could further enhance virtual experiences. Possibilities to integrate touch into digital and communication technologies are presently being intensively researched and evaluated^16,18,19^. When these technologies are matured, they could provide new multisensory experiences for humans to perceive in virtual (or remote) environments through digitalized tactile and kinematic information^10,20^, besides visual and auditory signals.

Building on Helmholtz’s classical theory (1857)^21^ and its modern Bayesian variants of perception^22–24^, we recently uncovered neural mechanisms underlying effects of multisensory congruence-based plausibility in modulating vibrotactile perception in VR contexts^25^. “Plausible experiences” in applied VR research commonly refer to correspondences between sensory events in virtually rendered contexts that confirm with prior expectations of the perceiver^26^. Specifically, we investigated cortical processes underlying the influences of bottom-up multisensory sensory congruency, top-down context-based expectation, and their interactions on vibrotactile perception in immersive virtual car-riding scenarios in young adults by using functional near infrared spectroscopy (fNIRS). The results of this previous study^25^ showed that cortical activities in the frontal and sensorimotor regions are modulated by multisensory contextual plausibility: vibrotactile stimulations congruent with the expected intensities given specific audio-visual information that were available in the respective virtual scenarios elicited greater brain activities than those which violated the expectations. Furthermore, individual differences in frontal activities that were observed under VR scenarios of high multisensory plausibility correlated negatively with expectancy violation costs in the sensorimotor cortex, indicating frontal regulation of multisensory processing in VR environments. Taken together, these results observed in young adults extended earlier findings on effects of contextual congruency in modulating perceptual processing in other modalities, including auditory^27^, taste^28,29^, and pain^30^ perception, to the sense of touch and to multisensory perception in VR environments. Insights into neurocognitive processes involved in the interplay between sensory congruence and contextual expectation that jointly affect the perceived plausibility of multisensory experiences in VR/AR are crucial to better inform the development of engineering solutions for plausible virtual multisensory experiences in digital environments. However, questions remain open as to how well these processes may still operate in older ages, considering particularly that different aspects of brain aging unfold gradually across the adult lifespan.

It is well-established that the volumes of several frontal-parietal regions^2,31^, which are involved in the processing contextual sensory expectations^25,32^, undergo clear declines during the adult lifespan. Furthermore, bilateral inferior frontal and superior parietal regions show weaker activity modulation by task demands with increasing age^4,33^. Aging also attenuates dopaminergic modulation^3^, which plays important roles in encoding and processing sensory signals as well as in attentional regulation of perception in several sensory modalities^34–36^. Focusing on tactile perception, other than age-related increases in tactile thresholds^37^ in the higher frequency ranges (i.e., 50–500 Hz), evidence from animal research showed that the firing rates of neurons in sensorimotor, parietal association, and frontal regions of Rhesus monkeys code the intensity and temporal features of vibrotactile inputs^38,39^. Moreover, dopamine is also involved in the detection of tactile stimuli and subjective tactile perception: specifically, the activities of dopamine neurons in the midbrain reflect uncertainty about the presence or absence of vibrotactile signals and the amplitudes of detected signals^40,41^. Taken together, considering gradual aging-related anatomical and functional declines of the frontal-parietal network and attenuated dopaminergic modulation, the aim of this study was twofold: (i) to investigate the extent to which cortical activities underlying the processing of multisensory congruency-based contextual plausibility in VR environments may still operate in older ages; and (ii) to identify the specifics of potential impairments in these processes in older adults. Answers to these questions would be helpful for developing age-inclusive digitalized VR-/AR-technologies to support cognitive and perceptual functions in older ages.

**Figure 1:**
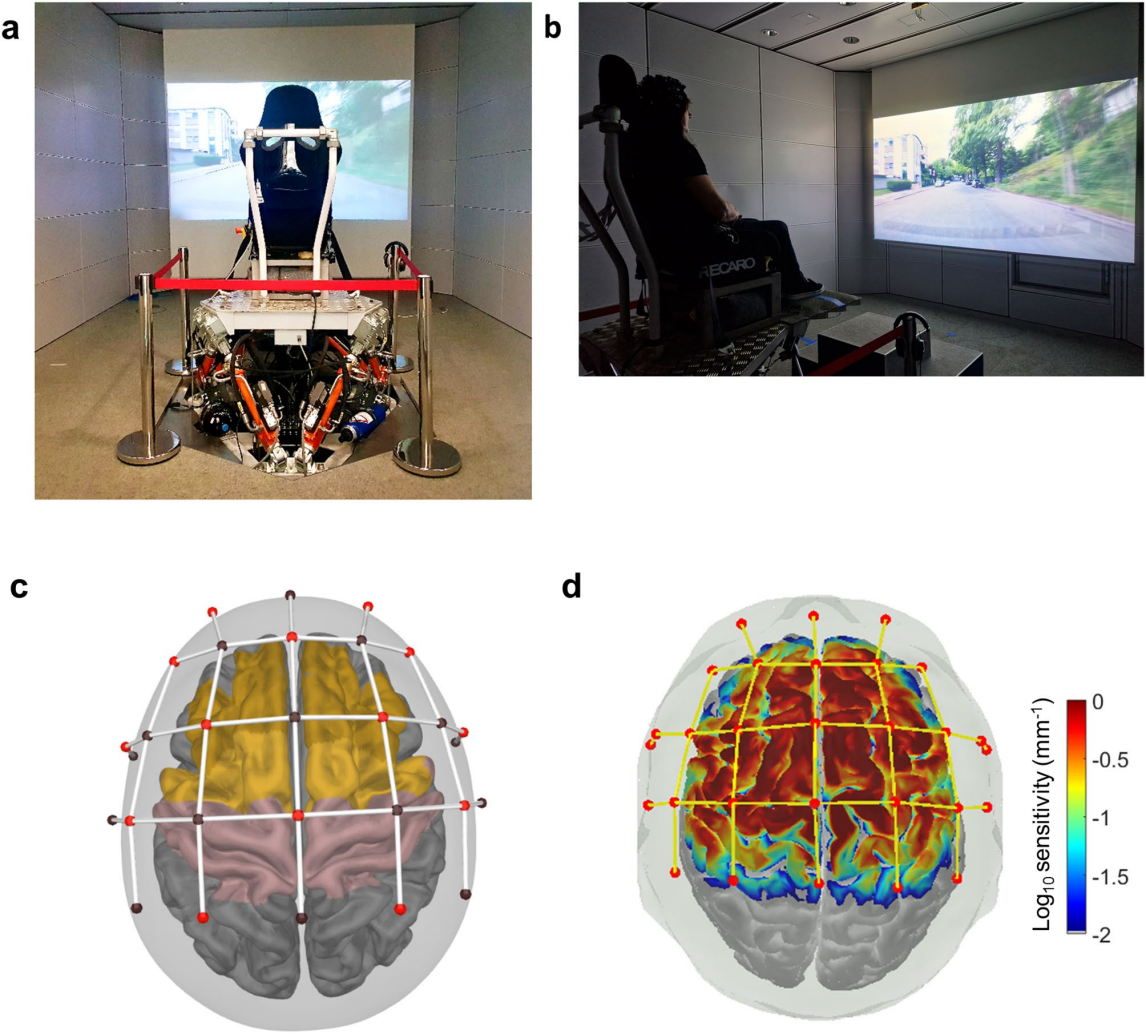
Experimental setup of the multisensory virtual-reality environment and fNIRS montage. (**a**) Vibrotactile stimulation was delivered by a seat mounted on a hydraulic platform and an electrodynamic shaker. (**b**) A participant wearing the NIRS cap is viewing audio-visual videos of road scenes from the perspective of a front seat passenger projected on a screen, along with the corresponding audio recordings reproduced with a wavefield synthesis system with 464 loudspeakers in the room which provides contextual information for the vibrotactile sensation. (**c**) The NIRS montage for this study covered the relevant brain regions of interest such as the dorsal lateral prefrontal cortex (dlPFC, dark yellow), premotor cortex with supplementary motor area (light yellow) and primary somatosensory cortex (pink). (**d**) The sensitivity profile of the NIRS montage shows that our montage is highly sensitivity to optical density changes measured by each source-detector pairs in the brain regions of interest (values are on the log_10_ scaled sensitivity values from 0.01 to 1.0).

We assessed cortical mechanisms of multisensory contextual plausibility in middle-aged and old adults (50–70 years) using fNIRS while they were exposed to the identical virtual scenarios of vehicle riding as those used in the abovementioned study of young adults^25^. Briefly, in the experimental paradigm several scenarios of virtual car riding sitting in the front row were created to manipulate the contextual plausibility of vibrotactile stimulation intensity in VR environments. The road scenes were carefully selected to be representative of daily experiences of vehicle riding. In each of the virtual scenarios, a supra-threshold vibrotactile stimulation of higher (e.g., 36 dB) or lower (e.g., 10 dB) intensity from a passenger seat was presented concurrently with the specific audio-visual information for a given road scene (see “Methods” section for details). The vibration was delivered through the seat that was securely mounted on a hydraulic platform with an electrodynamic shaker. The different audio–video scenes displayed views from the front passenger seat of a vehicle moving through road surfaces of different roughness (i.e., cobblestone, fine cobblestone, tarmac, and highway) that are, respectively, accompanied by congruent reproduced audio sound effects recorded from the corresponding road situations (Fig. 1a,b; see “Methods” section for details). Given previous evidence of brain regions relevant for vibrotactile and trimodal perception^42^, we used a montage with the NIRS sources and detectors covering the dorsolateral prefrontal (dlPFC), premotor (including the supplementary motor area) and the sensorimotor regions (Fig. 1c,d; see “Methods” section for details).

In accordance with the definition of plausible virtual experiences in VR research^26^, we operationalized contextual plausibility in the paradigm as the degree of conformity between the experienced multisensory congruence cues in VR and the participant’s expectations about the vibration intensity to be felt through the seat based on what would normally be expected for a given road type. Being a passenger riding in the front row of a car that moves through roads with different surface conditions are common daily experiences for most adults. Thus, according to Helmholtz’s^21^ classical and Bayesian theories of perception^22–24^, such prior knowledge based on past experiences can serve as a basis for expecting weaker vibrations from the passenger seat when audio-visual virtual experiences indicating a ride on smoother roads (i.e., quieter driving noise and visual scenes of smooth surfaces) or expecting stronger vibrations when confronted with audio-visual information of rough road situations (e.g., louder driving noise and visual scenes of rough surfaces). A vibration intensity that is congruent with the contextual multisensory expectation would yield a higher degree of perceived plausibility of the virtual experiences than one that is incongruent with (or violate) the expectation. While being on the virtual rides, middle-aged and old adults were asked to simply experience the situations and reflect about the plausibility of the vibrations felt through the seat. No explicit responses nor perceptual decisions were required during the main experiment. Subjective plausibility ratings of vibrotactile stimulations over a wider range of intensity levels were also obtained from the participants in a separate phase of the study (see “Methods” and “Results” sections for details).

To anticipate, the main findings show that, although cortical activities in frontal and sensorimotor regions are modulated by contextual plausibility in middle-aged and old adults, the effects are more constrained considering previous effects observed in young adults. In older ages, plausible vibrotactile stimulations which conformed with the participants’ expectations based on the available contextual audio-visual information of the respective virtual scenarios also elicited greater brain hemodynamic responses than stimulations of low plausibility. However, in older adults this effect was more circumscribed in that it was only present in virtual multisensory scenarios with extreme rough or very smooth road surface (i.e., cobblestone and highway, respectively). Furthermore, the effects of multisensory congruence-based plausibility in modulating cortical activities were positively correlated between brain regions in middle-aged and old adults. However, unlike previously observed in young adults, individual differences in frontal activities during scenarios of high contextual plausibility did not systematically correlate with plausibility violation costs measured in the sensorimotor cortex.

## Results

### Plausibility-based activity recruitments only observed in scenarios of extreme road scenes

We first evaluated the impacts of multisensory congruence-based plausibility on cortical activities when participants experienced vibrations of different intensity in virtual scenarios of road scenes displaying four different surface roughness (e.g., see an example in Fig. 1a,b; the videos, sound files, and digital files of vibrotactile stimulations for the virtual scenarios used in the study are available through the link provided in the Data and stimuli availability statement). Depending on the congruency or incongruency between the audio-visual scenes reproduced from the four different road types and the intensities (e.g., 10 dB vs. 36 dB) of vibrotactile stimulations, top-down multisensory contextual expectations and bottom-up multisensory congruence cues together resulted in scenarios of high or low perceptual plausibility.

As a manipulation check, the participants’ subjective plausibility ratings of vibrations across a wide range of stimulation intensities were obtained in a separate phase of the study (see “Methods” section). Results of the statistical test confirmed that vibrotactile stimulations in the high-plausibility scenarios were rated as significantly more plausible (mean ratings ranging from 62.06 to 79.13) than stimulations in the low-plausibility scenarios (mean ratings ranging from 17 to 51.26), *F*(1, 257) = 172.38,* p* < 0.0001, $\eta_{p}^{2}$ = 0.40. Of note, the effect size of subjective ratings of plausibility observed here in middle-aged and old adults is comparable to what was found in young adults ($\eta_{p}^{2}$ = 0.49) in an earlier study^25^. These results established a sound basis for evaluating effects of contextual plausibility at the cortical level and for interpreting effects of aging relative to earlier findings in young adults.

**Figure 2:**
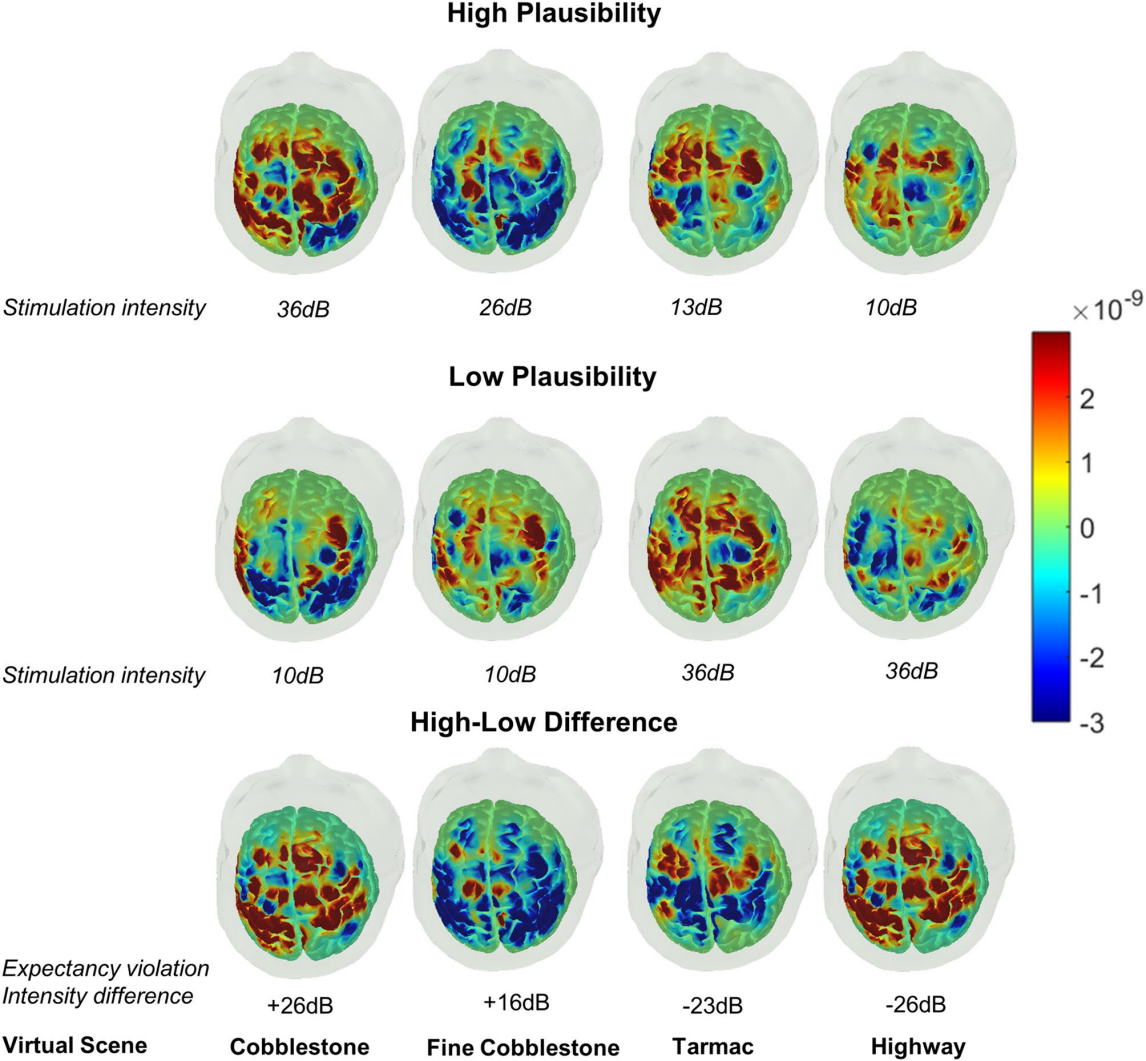
Topographical plots of HbO concentration levels across all channels for four road scenes at two plausibility levels and their differences. Columns represent different road scenes while the top, middle and bottom rows show HbO concentration levels for high plausibility scenarios, low plausibility scenarios and difference between high and low plausibility, respectively.

Cortical activities of participants (effective sample N = 33, 21 males, mean age = 62.36, SD = 6.01) were analysed using linear mixed-effects models (LMMs) of the fNIRS data. The LMMs have been increasingly used in psychological research because they allow more adequate modelling of data that are nested within participants by simultaneously considering variability within as well as across participants and multiple variables^43^. These models are also well-suited for analysing fNIRS data since haemoglobin concentrations from multiple channels are collected for a given participant in different experimental conditions^44^. The models used here included two experimental factors (type of road scene and plausibility) as fixed effects, whereas the NIRS channels nested with participants were included as random intercepts. For each of the participants, the number of observations was relatively large (i.e., 36 NIRS channels for 8 experimental scenarios), which resulted in large degrees of freedom. Consequently, the resulting values of $\eta_{p}^{2}$ would be numerically small given that they are computed from the values of the test statistics and the associated degrees of freedom^45,46^ (see “Methods” section for detailed information about participants, effective sample size, and derivations of degrees of freedom for the LMMs). The observed main and interaction effects are summarized in Fig. 2.

The overall analysis of the levels of oxygenated haemoglobin (HbO) concentration from all NIRS channels revealed significant main effect of road scene, *F*(3, 8106) = 10.52, *p* < 0.0001, $\eta_{p}^{2}$ = 0.0038 (note that measures of fNIRS reflect local positive or negative changes in haemoglobin concentrations relative to a baseline and do not reflect absolute concentrations; see “Methods” section for information about pre-processing procedures of fNIRS data). Although there was no significant main effect of plausibility, *F*(1, 8106) = 2.55, p = 0.11, $\eta_{p}^{2}$ = 0.0003, a significant scene x plausibility interaction, *F*(3, 8106) = 15.84, *p* < 0.0001, $\eta_{p}^{2}$ = 0.006, was observed. This 2-way interaction indicated a stronger effect of congruence-based plausibility on HbO in scenes with extreme rough surface (cobblestone, *t*(8106) = 5.017, 95% CI [0.0000003 0.000001], [*Bonferroni]-Adjusted p* < 0.0001, *d* = 0.21) and very smooth surface (highway, *t*(8106) = 3.183, 95% CI [0.000000009 0.000001], *Adjusted p* = 0.041, *d* = 0.13). For road scenes with intermediate surface roughness, a difference was only observed for the fine cobblestone scene, but in a reversed order, i.e., a weaker HbO activity in the high plausibility scenario than low plausibility scenario conditions,* t*(8106) = -3.559, 95% CI [− 0.00000106 − 0.000000069], *Adjusted p* = 0.01, *d* = -0.148. Together, these results indicated more constrained and less consistent effects of contextual plausibility on cortical activities in older adults (see text and Fig. S1 in Supplementary Materials for secondary analyses that directly compared the current data of middle-aged and old adults with published data of young adults^25^ using age-adjusted pathlength factor). We focus our report here on analyses of changes in HbO concentrations because of the higher signal-to-noise (SNR) ratio of these signals and their positive correlations with fMRI BOLD responses^47^ (see “Methods” section for further information). Nevertheless, results from analyses of deoxygenated haemoglobin (HbR) also showed significant effects of Scene, *F*(3, 8106) = 4.65, *p* = 0.003, $\eta_{p}^{2}$ = 0.0017, and Plausibility, *F*(1, 8106) = 19.27, *p* < 0.0001, $\eta_{p}^{2}$ = 0.0024 (see text and Fig. S2 in Supplementary Materials for more details).

### Effects of contextual plausibility on activities in frontal and sensorimotor regions

**Figure 3:**
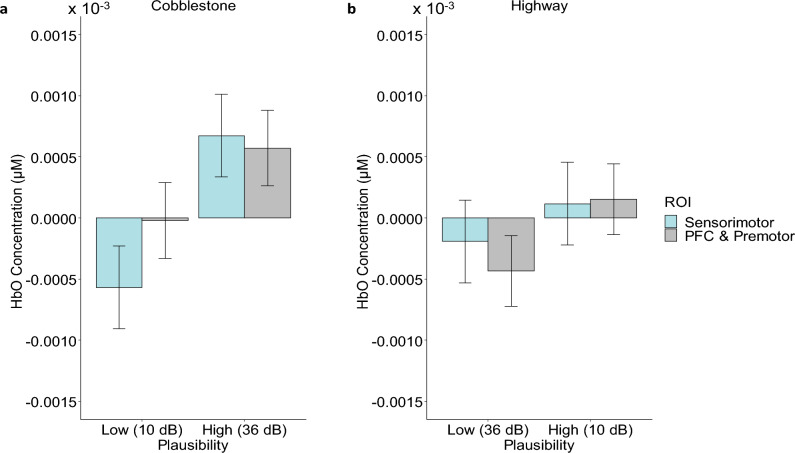
Marginal means of HbO concentration levels in the frontal and sensorimotor regions depicting the effects of congruence-based plausibility and cortical regions during scenarios with large expectancy violations. (**a**) cobblestone road scene and (**b**) highway road scene. Error bars represent ± 1 standard error of mean.

Following up the scene type x plausibility interaction reported above, separate analyses were conducted for the two extreme scenes, i.e., scene with extreme roughness (cobblestone) and smoothness (highway). These VR scenarios had a larger expectancy violation, which was experimentally defined by the intensity difference (+/− 26 dB) between the high and low plausibility conditions, than the scenarios with road scenes of intermediate roughness (with intensity difference of +16 or −23 dB; see Fig. 2 bottom panel). Within each of the two scenarios, the HbO concentrations in the sensorimotor cortex were compared against those in the frontal (dorsolateral prefrontal, premotor and supplementary motor cortex) regions which are known to implicate cognitive control processes, planning, conceptual expectancy^48,49^ and the integration between top-down congruency expectation and bottom-up congruence cues during multisensory perception^23,25,32^. The results (see Fig. 3) showed a significant main effect of plausibility in both scenarios (Cobblestone: *F*(1, 1157) = 30.41, *p* < 0.0001, $\eta_{p}^{2}$ = 0.03; Highway: *F*(1, 1157) = 7.44, *p* = 0.0065, $\eta_{p}^{2}$ = 0.006), but no significant main effect of brain regions in either scenarios (Cobblestone: *F*(1, 1125) = 2.05, *p* = 0.15, $\eta_{p}^{2}$ = 0.004; Highway: *F*(1, 1125) = 0.26, *p* = 0.61, $\eta_{p}^{2}$ = 0.0004). The brain region x plausibility interaction was only observed in the road scene with extreme roughness, Cobblestone: *F*(1, 1157) = 4.32, *p* = 0.038, $\eta_{p}^{2}$ = 0.004, showing plausibility-based modulation of cortical activity to be larger in sensorimotor cortex than in frontal region; whereas, no such interaction was observed in the scene of smooth road (Highway: *F*(1, 1157) = 0.497, p = 0.48, $\eta_{p}^{2}$ = 0.0004).

### Cortical effects of expectancy violation and multisensory congruency

Our experimental paradigm crossed scenes of rougher (e.g., cobblestone) or smoother (e.g., highway) roads with vibrotactile stimulations of higher or lower vibration intensities (e.g., 36 dB or 10 dB) to manipulate plausibility of the experienced vibration intensity. This allowed us to explore the nature of multisensory contextual expectancy in terms of negative or positive expectation violation costs in the sensorimotor cortex. To this end, we first compared the effects of a given vibrotactile stimulation intensity between scenarios of the highway and cobblestone scenes. Specifically, we computed the difference between HbO concentration in the sensorimotor cortex under the 10-dB stimulation in the cobblestone scene and that of the highway scene (cobblestone_Low Plausibility (10 dB)_ – highway_High Plausibility (10 dB)_). This difference reflects a negative expectancy violation cost, because 10-dB fails to meet the expected stronger vibration from the seat as what could be anticipated from seeing the rough cobblestone road in the reproduced scene-specific video and hearing the loud driving noise from the soundtrack. In the current study, middle-aged and old adults did not show a negative expectancy cost in the sensorimotor cortex, yielding a non-significant difference in HbO concentrations between the two conditions (*t*(1065) = -2.02, 95% CI [− 0.0000015 0.0000002], *Adjusted p* = 0.26, *d* = -0.14). Next, we computed the difference between HbO concentration in the sensorimotor cortex under the 36-dB stimulation in the highway scene and that in the cobblestone scene (highway_Low Plausibility (36 dB)_ – cobblestone _High Plausibility (36 dB_). This difference reflects a positive expectancy violation cost, since 36 dB exceeds the expected vibrotactile intensity from the car seat based on the scene-specific contextual audio-visual information of cruising on the highway. In this case, we did observe a significant effect, (*t*(1065) = 2.68, 95% CI [0.000000011 0.0000018], *Adjusted p* = 0.045,* d* = 0.19), indicating that under the same strong intensity of vibrotactile stimulation (36 dB), if the audio-visual information led to expectations of a weaker vibration, a lower level of activity was observed in the sensorimotor cortex despite the strong vibrotactile stimulation.

We next compared the effects of different vibrotactile stimulation intensities in the same scene as a function of multisensory congruency. In the scene of extreme smooth road surface (i.e., the highway scene), a higher HbO level was observed in the scenario with a lower stimulation intensity (10 dB) that was congruent with the expectation elicited by the audio-visual scene than a stronger (36 dB) vibrotactile stimulation which violated contextual expectancy. This effect was only observed in the frontal regions (dlPFC & Premotor: *t*(1157) = 2.66, 95% CI [0.000000004 0.0000012], *Adjusted p* = 0.047, *d* = 0.13), but not in the sensorimotor cortex (:*t*(1157) = 0.93, 95% CI [− 0.00000079 0.00000071], *Adjusted p* > 0.99, *d* = 0.07). As for the scene with extreme rough road surface (i.e., the cobblestone scene), we observed a higher HbO concentration for a stronger intensity of vibrotactile stimulation (36 dB) that was congruent with the expectations elicited by the audio-visual scene, as compared to a weaker (10 dB) vibrotactile stimulation which violated contextual expectancy in both frontal and sensorimotor regions (dlPFC & Premotor: *t*(1157) = 3.44, 95% CI [0.000000137 0.00000105], *p* = 0.0036, *d* = 0.155; Sensorimotor:* t*(1157) = 4.79, 95% CI [0.00000056 0.00000193], *p* < 0.0001, *d* = 0.32).

### Associations between expectation violation costs in frontal and sensorimotor regions

**Figure 4:**
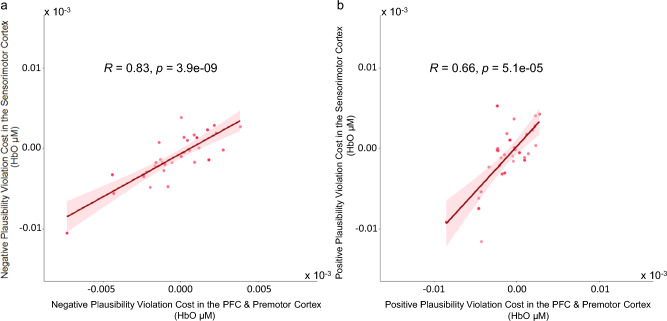
Scatterplots depicting positive correlations between expectancy violation costs in the frontal and sensorimotor regions. (**a**) Negative plausibility violation cost was computed as the difference in HbO concentration levels between cobblestone_10dB_ and highway_10dB_ scenarios (*n* = 32 old adults). (**b**) Positive plausibility violation cost was computed as the difference in HbO concentration levels between highway_36dB_ and cobblestone_36dB_ (*n* = 33 old adults). Shaded area represents 95% confidence interval.

We then conducted correlational analyses to examine potential associations between effects of multisensory contextual plausibility in frontal and somatosensory regions. To this end, we first computed expectancy violation cost scores for each participant for each of the two regions of interest (see “Methods” section for details of cost calculation and measures to address potential confounds of individual differences in the SNR of NIRS signals). In addition, the expectancy violation costs computed here used each participant’s HbO concentrations in conditions without expectancy violations as baselines to account for potential individual differences in baseline cortical activities. Results from the correlational analyses showed that those participants who showed a stronger expectation violation effect in the frontal regions also showed a stronger effect in the sensorimotor cortex, irrespective of whether it was positive (*rho*_*cost*_ (31) = 0.66, *p* < 0.0001,* N* = 33) or negative (*rho*_*cost*_(30) = 0.83, *p* < 0.0001, *N* = 32) expectancy violation costs (see Fig. 4).

### No relation between frontal activities in high-plausibility scenarios and sensorimotor violation costs

**Figure 5:**
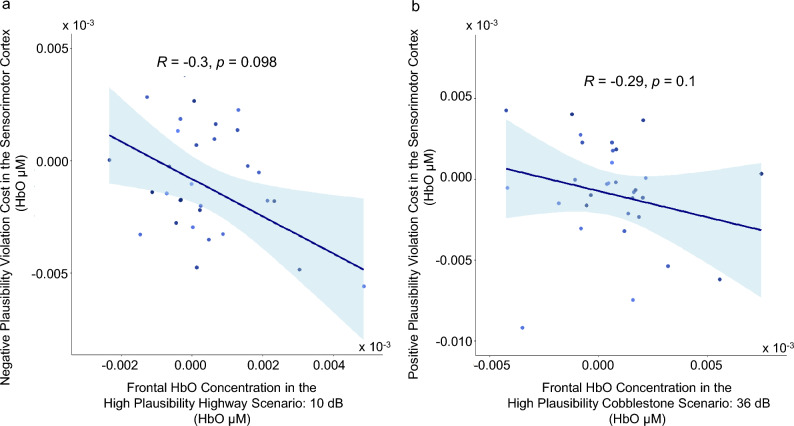
Scatterplots depicting the lack of systematic relations between frontal HbO concentration under high plausibility scenarios and expectation violation costs in the sensorimotor cortex. (**a**) Scenario of high plausibility with 10-dB vibrotactile stimulation (*n* = 32 old adults). (**b**) Scenario of high plausibility with 36-dB vibrotactile stimulation (*n* = 33 old adults). Shaded area represents 95% confidence interval.

Lastly, we examined the correlation between frontal HbO concentration levels assessed in scenarios of high multisensory plausibility (i.e., cobblestone road scene with 36-dB vibrotactile intensity or highway road scene with 10-dB vibrotactile intensity) and the magnitudes of expectancy violation costs in the sensorimotor cortex. No significant correlations in middle-aged and old adults could be found in either of the two scenarios with extreme road scene (Fig. 5. cobblestone road scene with 36-dB vibrotactile stimulation: *rho*_*cost*_(31) = -0.288, *p* = 0.10, *N* = 33; highway scene with 10-dB vibrotactile stimulation: *rho*_*cost*_(30) = -0.29, *p* = 0.098, *N* = 32). Together, results of these correlational analyses revealed that, in older ages individual differences in plausibility elicited frontal activities did not vary systematically with the magnitudes of expectancy violation costs in the sensorimotor cortex.

## Discussion

In this study, we aimed to investigate whether cortical processes underlying multisensory plausibility modulation of vibrotactile perception are still functional in middle-aged and old adults and to identify which aspects of these processes may differ from those previously observed in young adults. Brain hemodynamic responses were assessed using fNIRS while participants experienced vibrotactile stimulations of different intensities that were either in accord with or violate multisensory expectations in immersive virtual multisensory VR vehicle riding scenarios. Brain processes underlying tactile perception that are associated with the commonly considered plausibility principle of virtual realism in applied VR research^26^ were recently revealed in young adults^25^. These processes operated through interactive effects of multisensory congruence cues and contextual expectations^22,23,27–29,50^. Crucially, cortical activities in frontal regions (frontal, premotor and supplementary motor) known to implicate top-down contextual expectations^32,51^ and in sensorimotor regions underlying somatosensory and motor processing^52,53^ were modulated by manipulating contextual plausibility: VR scenarios with high contextual plausibility elicited greater cortical activities than scenarios with low plausibility. Moreover, multisensory plausibility elicited cortical activities in frontal regions were associated with modulations of cortical activities in the sensorimotor cortex in terms of expectancy violation costs, suggesting frontal contextual expectation-based modulation of tactile representations in the somatosensory cortex^25^.

Keeping in mind the abovementioned findings in young adults, the current results found in middle-aged and old adults are summarized and discussed in the following aspects. First, cortical activities underlying tactile perception are still responsive to multisensory congruence-based plausibility in VR environments in older ages; however, the effects are more circumscribed relative to those found in young adults. Plausibility-dependent recruitments of cortical activities in middle-aged and old adults were only substantially present in VR scenarios with road surfaces that are at the end of the roughness–smoothness spectrum of the stimuli used in the current paradigm (i.e., scenes with cobblestone road or highway, respectively). In these two scenarios, contextual audio-visual information coupled with manipulating the intensity of vibrotactile stimulations resulted in larger expectancy violations than the other two road scenes of intermediate surface roughness. Processing smaller differences in the expected vibration intensities in scenes with moderate roughness (fine cobblestone) or smoothness (tarmac road) would require finer sensory and perceptual sensitivity, which makes these two scenarios more demanding. This pattern of results is partly consistent with findings from fMRI^4,33^ and fNIRS studies^54^ of aging, which showed attenuations of task demand related recruitments of cortical activities in several frontal and parietal regions in older adults.

The limited effect of contextual plausibility can also be considered from a different level. Findings from animal research indicate that neurons in sensorimotor, parietal association, and frontal regions code the intensities and temporal features of vibrotactile signals^38,39^: Furthermore, activities of midbrain dopamine neurons reflect uncertainty about the presence or absence of vibrotactile signals and the amplitudes of detected signals^40,41^. Thus, aging-related declines in striatal dopamine modulation either via dopamine D2 receptor or transporter^3^,^55^ may contribute to noisier perceptual representations as suggested by earlier neurocomputational simulations^56^. Noisier perceptual representations can render effects of contextual plausibility on cortical activities in older ages to be weaker and only present in conditions with large expectancy violations. Compared to previous findings in young adults showing that violation-related attenuation of activities in sensorimotor cortex is relatively independent of the direction (negative or positive) of expectancy violation^25^, the current results only revealed such an effect with respect to positive, but not for negative, expectancy violation cost. When middle-aged and old adults experienced a strong vibrotactile stimulation (36 dB) that exceeded (i.e., positive expectancy violation) expectations based on the audio-visual information in the VR scenario (scene with highway), activities in the sensorimotor cortex were attenuated relative to when the same strong intensity would be experienced in a plausible context (i.e., scene with the cobblestone road). However, no such effect of activity modulation was observed for negative expectancy when older adults experienced a weak stimulation (10 dB) that was relatively much weaker than what they would have expected based on the audio-visual information of the VR scenario (scene with cobblestone road). Thus, the lack of negative expectancy cost in middle-aged and old adults in this case may also, in part, be attributed to their declined sensory processing (e.g., increased vibrotactile thresholds^37^), which makes cortical sensory effects elicited by low-intensity signals weaker and, consequently, difficult to discern their modulations by contextual expectations.

In terms of associations between multisensory expectancy violation costs in the frontal and sensorimotor regions, the effects of expectancy violation-based attenuations (costs) of cortical activities in these regions are significantly correlated in middle-aged and old adults independent of directions of the expectation violation (Fig. 4), very much in line with previous findings observed in young adults^25^. Going beyond the current VR paradigm, this finding is also consistent with previous results concerning the visual modality. Specifically, effects of contextual facilitations of cortical recruitments in brain regions of visual object processing and in hippocampal areas of high-level memory-based scene processing were found to be correlated^57^. Thus, the current finding indicates that sensitivities to multisensory congruence-based recruitments of cortical activities in regions implicating contextual expectations and sensory processing still reflect systematic individual differences in older ages. However, unlike previous findings observed in young adults^25^, frontal activities during high-plausibility scenarios did not correlate with expectancy violation-based modulation of activities in sensorimotor regions in middle-aged and old adults (Fig. 5). Given aging-related decline of the frontal lobe^58^ at the anatomical^2,31^ and neurochemical^3,55,56,59^ levels, this result may reflect that, frontal activities known to support flexible and context-based sensory filtering^60,61^ become weaker in middle-aged and old adults. Thus, in older ages plausibility-elicited activities in frontal and premotor regions may not systematically relate to individual differences in sensory expectancy violation costs measured by activity attenuations in the sensorimotor cortex. Given that the observed effects of contextual plausibility are already more circumscribed in the current sample of middle-aged and old adults (50 – 70 years) relative to young adults (cf. Kang et al., 2022 & Fig. S1 in Supplementary Materials), sensory intensity as well as multisensory congruency (modality- and temporal-based) need to be even more carefully adjusted when designing sensory augmentation technologies for populations of these and even older ages (e.g., > 70 years).

Taken together, current results suggest that processes of multisensory congruence-based recruitments of cortical activities underlying contextual expectancy modulation of perception in several other modalities^27–30^ may also be affected by aging. The Bayesian inference framework^22–24^ of perception which formalize contextual expectations as statistical priors about the multisensory environments would be a suitable approach to more specifically model aging-related constraints on effects of contextual plausibility. Combining formal modelling with pharmacological studies that vary levels of dopamine agonists^62,63^ (e.g., such as L-DOPA) to systematically study multisensory contextual expectation-based modulation of perception in different modalities in future research could shed further lights on how these mechanisms change in older ages. The current findings might also inform new engineering solutions for generating plausible virtual multisensory experiences. For instance, future research may explore approaches for also integrating neural correlates of contextual expectations to improve generative models for synthesizing plausible vibrotactile stimulations^64^, which currently are only based on subjective ratings of perceptual experiences. Given current results, a higher degree of multisensory congruency in virtual scenarios of multimodal stimulations with higher sensory intensities need to be considered when designing age-inclusive VR and digital technologies^10^ to support daily cognitive, perceptual, and sensorimotor functions of the growing aging populations worldwide. Although in the current work we only focus on effects of multisensory contextual plausibility on vibrotactile perception, previous research suggests broader implications of haptic augmentation technologies for enhancing postural control^65,66^, locomotion^66,67^, and hand movments^68^ that are highly relevant for rehabilitation therapies in the elderly and patients for whom sensorimotor functions are compromised either due to gradual declines^69^, neuroimmune degenerations, or acute brain lesions^70^. Furthermore, besides the here observed effects of modality-based congruence on perceived plausibility of vibrotactile augmentation, temporal synchronicity also influences the integration of haptic inputs with other sensory signals in virtual environments^71–73^. Thus, future research also needs to investigate effects of temporal congruency on the plausibility of multisensory augmentations that aim to support perceptual and sensorimotor functions of the aging populations either in digitalized virtual environments or in remote environments via multimodal digital transmissions^74^.

## Methods

### Participants

Forty-three adults in the age range from 50 to 70 years (mean age = 62.36 years, SD = 6.01, 21 males) participated in the study. As such, the sample had a broader and more continuous age range that also included middle-aged adults, a population that is much rarely studied than the already retired population. Since cognitive and perceptual declines tend to progress gradually across later periods of the adult lifespan, we recruited a sample that also included the middle age. All participants had normal or corrected-to-normal vision. Forty-one participants were right-handed as measured by the Edinburgh handedness Inventory^75^. Informed consent was provided by all participants prior to the study. They were compensated for their participation. The study was approved by the Ethics Committee of the Technische Universität Dresden (SR-EK-5012021) and have been performed in accordance with the Declaration of Helsinki.

### Multimodal stimuli

The stimuli used in this study consisted of naturalistic reproductions of daily car-riding situations. Video recordings of common vehicle driving situations on four different types of road surfaces (cobblestone, fine cobblestone, tarmac, highway) at a speed of 50 kph were captured from the perspective of the front seat using a Canon EOS 600D Camera with an optical image stabilizer lens. Two B&K 2671 microphones were attached to the head rest of the seat to record the sound effects heard at the ear of the passenger. Meanwhile, a seat pad accelerometer (B&K 4515B) and a Kistler 8305B10 sensor were, respectively, used to record vertical seat vibrations and low frequency vibrations.

All these naturalistic multimodal scene recordings were used as virtual multisensory scenarios in the experiment. The original audio-visual recordings recorded from the scenes were used as stimuli in the scenarios. Synthesized vibrations (sinusoidal, amplitude modulated sinusoidal, bandlimited white Gaussian noise) similar to previous studies^25,76^ were used. These synthesized vibrations were rated to be of the same levels of plausibility as the recorded vibrations^76^. Since different levels of vibration intensity could affect the perceived plausibility of a given audio-visual scene^77^, the acceleration level of the synthesized vibrations for each of the road scenes in the subjective rating phase of the study was set to 10, 13.25, 16.50, 19.75, 23, 26.25, 29.50, 32.75 and 36 dB in sensation level relative to the perceptual threshold of whole-body vibration^78^. The maximum acceleration level was selected due to constraints imposed by the reproduction system, whereas the minimum acceleration level was selected to be above the perceptual threshold to ensure perceivability. The parameters of the synthesized vibrations at different sensation levels used for the virtual scenarios of the main experimental task are shown in Table 1.

**Table 1:** Parameters of the synthesized vibrations at different sensation levels (parameters which are undefined for the respective signal type are marked by "NA").

Road Scenes	Sensation level	(centre-) frequency	Bandwidth	Modulation frequency
Cobblestone	36 dB	26	50	0
Fine Cobblestone	26.25 dB	9	NA	NA
Tarmac	13.25 dB	155	NA	15
Highway	10 dB	7	NA	2

### Experimental design

The plausibility of vibrotactile stimulations in virtual multisensory road scenes were experimentally manipulated by crossing the vibration levels with four road scenes of varying surface smoothness. The sensation levels of vibrotactile stimulation in the high and low plausibility scenarios for the four road scenes in the main experimental task are shown in Table 2. Thus, the experimental design of the main task was a 2 × 4 (plausibility x scene) within-subject design. Each of the plausibility (high vs. low) by scene (4 road scenes) combinations were repeated 15 times, resulting in a total of 120 trials. The presentation order of virtual scenarios was randomized. The participants were only asked to passively reflect about the plausibility of the vibrotactile stimulation in each of the eight virtual car-riding scenarios. They were not required to respond nor make any perceptual decision explicitly.

**Table 2:** Vibration levels of the passenger seat in high plausibility and low plausibility scenarios in the main experimental task for each of the four scenes.

Road Scenes	High Plausibility (dB)	Low Plausibility (dB)
Cobblestone	36	10
Fine Cobblestone	26.25	10
Tarmac	13.25	36
Highway	10	36

Other than the main experimental phase, a separate subjective rating phase was also included in which participants were asked to rate the plausibility of vibrotactile stimulations across 9 intensity levels (10, 13.25, 16.50, 19.70, 23, 26.25, 29.50, 32.75, 36 dB), which were paired once with each of the four road scenes. This resulted in a total of 36 scenarios for subjective ratings in this phase. This allowed us to assess incremental changes in subjective rating in response to a wide range of vibration levels. Participants were asked to verbally rate the plausibility levels on a quasi-continuous Rohrmann scale^79^ which ranged from 0 to 100 with equidistant anchors at 0 (“not at all” plausible), 25 (“slightly” plausible), 50 (“moderately” plausible), 75 (“very” plausible) and 100 (“extremely” plausible)^76,77^.

### Experimental Setup

The multimodal virtual audio-visual-tactile scenarios described above were presented in an immersive multimodal measurement laboratory^76^. A Full-HD projector was used to achieve optical reproduction on a screen with a diagonal of 300 cm and at a distance of 340 cm from the participant. A wavefield synthesis system consisting of 464 individually controllable speakers were used to achieve naturalistic acoustic reproduction and recreate the wavefield produced by the original recorded sound source. Contrary to acoustic reproduction using headphones, this system does not suffer from shortcomings such as in head localization of sounds which could potentially affect the immersive experience of participants. Moreover, unlike stereo setups, it also does not depend on phantom source effects. Hence, our audio reproduction of road scene recordings was not affected by any unwanted changes in perceived direction of sounds that could result from head movements. Seat vibrations were reproduced by two systems to include all perceivable frequency range of everyday life vibrations ranging from 1 to 500 Hz. A hydraulic motion platform was used to present low frequency vibrations below 15 Hz while an electrodynamic shaker attached to the seat surface was used to present high-frequency vibrations. In order to account for individual differences in body weight or height, vibration reproduction was calibrated for each participant and the transfer function was measured before the start of the experiment. It was then subsequently compensated with an FIR filter^80^ to ensure identical vibration reproduction for every participant. The multimodal reproduction system which was used as the immersive virtual environment in our experiment is displayed in Fig. 1 (Fig. 1a,b).

### Study procedure

Prior to the start of the main experiment, participants’ basic cognitive speed and verbal ability were measured using two commonly used psychometric tests, i.e., the Identical Pictures Test^81^ and the Spot-the-Word Test^82^. Furthermore, individual differences in experiences with computer/video games were assessed with a 6-point scale, with “1” indicating “not at all”, “2” indicating “less than 30 min per day”, “3” indicating “ 30 min to 1 h per day”, “4” indicating “1 to 2 h per day”, “5” indicating “2 to 4 h per day” and “6” indicating “more than 4 h per day”. After these assessments, all participants took part in two phases of the experiment, i.e., the main experimental phase and the subjective rating phase. The order of these two phases were counterbalanced across participants. In the main experimental phase, an event-related study design was implemented to measure brain hemodynamic responses using fNIRS. Participants passively experienced the multisensory virtual scenarios of car rides on four different road surfaces with varying intensities of vibrotactile stimulations (see Table 2) from the passenger’s seat (see Fig. 1a,b). Each of the eight virtual scenarios was presented for 4 s, whereas the inter-trial interval (ITI) was jittered according to the following formula:

$${\text{ITI}} = T_{{{\text{Load}}}} + T_{{{\text{Scene}}}} + T_{{{\text{Transition}}({\text{pseudorandom}})}} + T_{{{\text{Random}}({\text{Geometric}})}} .$$

In the equation above, *T*_Load_ denotes the time taken for a given scene to load, *T*_Scene_ denotes the scene duration (4 s), *T*_Transition(pseudorandom)_ denotes the time taken to progress to the next scene and *T*_Random(Geometric)_ denotes a random number generated from a geometric distribution. The mean ITI across all trials for all participants ranged from 14.82 s to 19.39 s (mean = 16.58 s).

### fNIRS data acquisition

A continuous-wave, battery-operated fNIRS system, i.e., the NIRSport 2(NIRx Medical Technologies, LLC, USA), was used to measure the concentrations of oxygenated (HbO) and deoxygenated (HbR) haemoglobin with two distinct wavelengths (i.e., 760 and 850 nm) at a sampling rate of 4.98 Hz. A MATLAB-based toolbox, known as the fNIRS Optodes Location Decider^83^ (fOLD v2.2), was used to design the NIRS montage using the standard 10–10 system. The Brodmann anatomical atlas was used to localize optodes to the brain regions of interest, such as the dorsolateral prefrontal cortex (BA 9), premotor and supplementary motor cortex (BA 6), primary somatosensory cortex (BA 1, BA 2, BA 3) and primary motor cortex (BA 4). To ensure full coverage of these regions, source optodes were positioned at AF3, AF4, F3, F2, F4, FC5, FC1, FC2, FC6, C3, Cz, C4, CP1, CP2, while detector optodes were positioned at AFz, F1, F2, FT7, FC3, FCz, FC4, FT8, C5, C1, C2, C6, CP3, CPz, and CP4 (see Fig. 1c for the montage). This montage yielded high sensitivity in covering the regions of interest as shown in the sensitivity profile (Fig. 1d). The sensitivity profile is based on changes in the absorption coefficients and provides information about whether the montage is sensitive to measurement of optical density changes for each of the source-detector pairs.

In summary, the fNIRS montage consisted of 36 long source-detector separation channels (i.e., 25 channels over the prefrontal cortex and premotor regions, 11 channels over the motor and somatosensory cortex) with an inter-optode distance of approximately 30 mm that was maintained with the help of linked optode holders. Short-separation channels were not used in this study since whole body vibrations might lead to artifactual short-distance measurements that could add noise into the signals of interest if such channels were added^84^. Instead, we removed unwanted physiological confounds by using principal component analysis^85^.

### fNIRS data pre-processing

Among the 43 participants who took part in the study, fNIRS data from 5 participants could not be obtained due to technical difficulties (e.g., hardware malfunction or not saved triggers). Pre-processing was conducted using Homer3^86^ (BUNPC) while visualization was conducted using AtlasViewer Toolbox^87^. At pre-processing, bad channels were identified and excluded using the hmrR_PruneChannels function with a SNR threshold criterion of 6.67 (equivalent to coefficient of variation (CV) = 15%; SNR = 1/CV*100). Datasets which consisted of more than 25% of bad channels (i.e., CV less than 15%^88,89^) were excluded from further analysis (*N* = 2). Raw signals of light intensity were then converted into changes in optical density using the hmrR_Intensity2OD function. Then, motion artefacts were identified as optical density units greater than an amplitude of 0.3 over half a second and marked for one second by using the hmrR_MotionArtifactbyChannel function. Outliers due to motion artefacts which were identified as wavelet coefficients that exceeded 1.5 times of the interquartile range were corrected using the hmrR_MotionCorrectWavelet function^90^ which typically reduces motion artefacts up to 93% of cases^91,92^. However, trials which still contained motion artefacts that could not be corrected were rejected using the hmrR_StimRejection function in the time range of 2 s before stimulus onset to 4 s post stimulus presentation. Datasets containing more than 20% of rejected trials were also removed from the analyses (*N* = 3). Therefore, the final effective sample for fNIRS data analyses contained data from 33 participants.

As the next step, a low-pass filter with a cutoff of 0.5 Hz^93^ was applied to remove high-frequency components (e.g., instrument noise) but retain signals of interest. However, this frequency range may still include physiological artefacts such as those induced by respiration (~ 0.3 Hz) and spontaneous oscillations in arterial blood pressure known as Mayer waves (~ 0.1 Hz). Thus, principal component analysis (hmrR_PCAFilter, nSV = 1.0) was applied to mitigate these physiological confounds and interferences from superficial layers of the scalp, skin and skull^94,95^. Although no short-separation channels were used, principal component analysis could yield results comparable to that of using short source-separation channels^85^. Next, the pre-processed and filtered optical density was converted into hemodynamic concentration values based on the modified Beer-Lambert Law by using the hmrR_OD2Conc function with age-dependent partial pathlength factor (PPF). To this end, we first calculated age-dependent differential pathlength factor (DPF)^96^ based on the mean age of the participants. Afterwards the DFP was then converted into PPF (PPF = 1/60 * DPF). Accordingly the DPF and PPF used for the current sample were: DPF_760nm_ = 7.17, PPF_760nm_ = 0.12, DPF_850nm_ = 6.10, and PPF_850nm_ = 0.10.

The hemodynamic response function (HRF) was estimated with a general linear model (GLM) which used the ordinary least squares function^97^ and modelled using consecutive sequence of Gaussian functions with mean and standard deviation of 0.5 s (glmSolveMethod = 1, idxBasis = 1, paramsBasis = [0.5, 0.5]). The regression time used was between − 2 to 12 s, whereas the pre-stimulus time of 2 s was used for baseline correction to account for intra- and inter-individual differences in cerebral oxygenation over time. A third-order polynomial fit was used to account for baseline drift^98,99^. The means of baseline-corrected concentrations of HbO and HbR between 1 to 5 s post-trial onset were computed, respectively, for further statistical analyses. This time window was selected because the stimulus duration was 4 s and the peak of the HRF typically occurs around 4 to 5 s. We only report the results of HbO concentrations because HbO has been shown to be more reliable than HbR^100,101^.

In order to visualize the cortical topography of brain activity, an atlas head model was registered using digitized points of sources and detectors to allow for a more accurate estimation of the location of cortical activation. From the measured changes in optical density and sensitivity profile (i.e., forward matrix), it is possible to perform an image reconstruction of changes in the absorption coefficient in the cortex^87^. Thus, the inverse problem can be solved by inverting the forward matrix. As such, image reconstruction can be accomplished with the following equation:

$${\mathbf{x}} = {\mathbf{A}}^{{\text{T}}} ({\mathbf{AA}}^{{\text{T}}} +\uplambda {\mathbf{I}})^{{ - {1}}} {\mathbf{y}}.$$

In the equation above, **x** denotes the spatial distribution of HbO or HbR absorption perturbation, **A** denotes the sensitivity profile (the forward matrix) of the registered atlas obtained using Monte Carlo photon migration simulation, λ denotes the scalar regularization parameter, **I** denotes the identity matrix, while **y** denotes the measurement vector which was provided as optical density changes in the data.

### Analyses using LMMs

The LMMs were used to analyze behavior rating and fNIRS data using the lme function from the nlme package in R RStudio (R-4.1.1). In LMMs the degrees of freedom of test statistics depend on the complexity of the model structure involving random effects and the approximation methods used to derive the degrees of freedom of the model. We used the containment method for estimating degrees of freedom as provided in S^102^ or SAS^103^; this method takes into account the hierarchical (nested) structure of the data. The partial eta squared ($\eta_{p}^{2}$), one of the indicators of effect sizes^104^, can be computed from the *F-*statistic and its associated degrees of freedom^45,46^ (for discussions about relations between degrees of freedom and threshold values of test statics in general see 44). Depending on the degrees of freedom, when using LMMs with complex variance–covariance structures, the values of partial eta-squared ($\eta_{p}^{2}$) tend to be small and may not be interpretable using the usual ranges that are applicable for less complex models, e.g., $\eta_{p}^{2}$ = 0.01 (small), $\eta_{p}^{2}$ = 0.06 (medium), $\eta_{p}^{2}$ = 0.14 (large). Post-hoc analyses were conducted with Bonferroni correction for multiple comparisons using the emmeans package in R^105^. For these post-hoc analyses, adjusted *P*-values were reported along with Cohen’s *d* as a measure of effect size with the following interpretation: *d* = 0.2 (small), *d* = 0.5 (medium), *d* = 0.8 (large).

### Subjective plausibility ratings of scenarios of different vibrotactile sensation levels

Participants’ subjective ratings for each of the four road scenes across 9 vibrotactile sensation levels (10, 13.25, 16.50, 19.75, 23, 26.25, 29.50, 32.75, and 36 dB) were obtained. Indeed, from the plausibility ratings for all four scenes, participants rated the vibrotactile stimulations to be more plausible in the scenarios which were defined to be of high plausibility (mean ratings ranging from 62.05 to 79.13) than in the low plausibility scenarios (mean ratings ranging from 17 to 51.26). For each scene, we calculated the plausibility ratio for each participant by dividing the difference between high and low plausibility ratings by the ratings in the low plausibility scenarios. However, because the ratings ranged from 0 to 100, a value of 1 was added to the denominator, resulting in the following formula: (Rating_high_ − Rating_low_)/(Rating_low_ + 1). Values that were 3 standard deviations away from the mean were identified as outliers and excluded from the analyses. The LMMs were also used to analyze participants’ subjective plausibility ratings with Scene (Cobblestone, Fine Cobblestone, Tarmac, Highway) and Plausibility (Low, High) as within-subjects fixed-effects and participants as random-effects.

#### Overall fNIRS neural activity during virtual scenarios of different contextual expectancies

The fNIRS data was analyzed using Matlab R2018b and RStudio (R-4.1.1). We used LMMs with maximum likelihood estimation to analyze the changes in HbO concentrations using the lme function from the nlme package in R. Given the large number of observations (36 NIRS channels for each of the 8 experimental scenarios) that were nested within the 33 random intercepts (one parameter for each participant) the resulting degrees of freedom were large in our models. The LMMs reported in this study were calculated with Scene (cobblestone, fine cobblestone, tarmac, highway) and Plausibility (low, high) as within-subjects fixed-effects; while participants were included as random intercepts with NIRS channels nested into participants in order to take into account variability in haemodynamic concentration changes across the channels^89^.

#### Scene- and region-based analyses

In order to investigate how cortical activities are modulated by contextual expectancy during extreme smooth and rough road scenes of low (10 dB) and high (36 dB) levels of vibrotactile stimulation, we focused our analyses on the Highway and Cobblestone scenes which has the largest sensation level difference between the least and most plausible scenarios. For each of the two scenes, the LMMs were used to analyze cortical activity in the brain regions of interest, i.e., the dlPFC regions (frontal attentional control and action planning) and the sensorimotor regions (somatosensory and motor) and Plausibility (High, Low) as fixed-effects and the NIRS channels nested in participants as random-effects. In order to compare how the perceptual representations of vibrotactile stimulation is modulated by contextual expectancy, the LMMs were set up with Intensity (10 dB, 36 dB) and Scene (Highway, Cobblestone) as fixed-effects and participants (with channels nested into participants) as random effects.

#### Frontal-sensorimotor correlations

To analyze the relationships between expectancy violation costs in the frontal regions and expectancy violation costs in the sensorimotor regions, we computed the negative and positive expectancy violation costs in the following way. Negative plausibility violation cost was computed by subtracting HbO concentration in the Highway_High Plausibility_ condition from HbO concentration in the Cobblestone_Low Plausibility_ condition (cobblestone_Low Plausibility (10 dB)_ − highway_High Plausibility (10 dB)_), whereas positive plausibility violation cost was computed by subtracting HbO concentration in the Cobblestone_High Plausibility_ condition from HbO concentration in the Highway_Low Plausibility_ condition (highway_Low Plausibility (36 dB)_ − cobblestone_High Plausibility (36 dB)_). The average HbO concentration changes across all channels in the dlPFC and premotor regions (frontal attentional control and action planning) as well as the sensorimotor regions (somatosensory and motor) were calculated for the correlational analyses. To avoid spurious correlations, outliers that were 3 standard deviations away from the mean were excluded (*N* = 32 for the correlation between negative plausibility violation costs in frontal regions and plausibility violation costs in the sensorimotor regions (Fig. 4); *N* = 32 for the correlation between negative plausibility violation cost in the sensorimotor cortex and average HbO concentration of the high plausibility highway scenario for the frontal regions (Fig. 5)). Pearson’s correlation was used for correlational analyses. However, Spearman’s correlation was used if the data was not normally distributed as indicated by Shapiro–Wilk test of normality and QQ-Plot (qqPlot, ‘car’ package).

#### Age-related differences in cortical activities under different contextual expectancies (in Supplementary Information)

To assess if there are age-related differences in cortical activities in car-riding scenes of different contextual expectancies, we combined the fNIRS data from the current study with middle-aged and old adults and a previous study^25^ with young adults in a secondary analysis. To allow for an age-related comparison, the analyses from the previously published young adults data were adjusted with age-dependent partial pathlength factor (PPF) based on the mean age of young adults (mean age = 23.86, DPF_760nm_ = 6.12, PPF_760nm_ = 0.10, DPF_850nm_ = 5.06, PPF_850nm_ = 0.08) while the middle-aged and old adults data were adjusted with age-dependent PPF based on the mean age of the current sample (mean age = 62.36, DPF_760nm_ = 7.17, PPF_760nm_ = 0.12, DPF_850nm_ = 6.10, PPF_850nm_ = 0.10). We then conducted analyses using a LMM with Scene (Cobblestone, Highway) and Plausibility (Low, High) as within-subjects fixed-effects, Age (Young Adults, Old Adults) as the between-subjects fixed-effect and participants (with NIRS channels nested in participants) as random-effects on HbO concentrations. A separate control analysis that used individual differences in computer/video gaming experiences as a covariate in the LMM was also conducted.

### Supplementary Information

Supplementary Information.

## Data Availability

The stimuli, data and code used for analyses in this manuscript can be found in the following Open Science Framework repository link: https://osf.io/625qn/?view_only=2f92087e7e6c42a6bf69d9aa507805f7
