# Supplementary material for: Cortical processes of multisensory plausibility modulation of vibrotactile perception in virtual environments in middled-aged and older adults
Source: Sci Rep. 2024 Jun 11;14:13366. doi: 10.1038/s41598-024-64054-z (PMC11166973; doi:10.1038/s41598-024-64054-z)
Supplement: Supplementary file 1 — Supplementary Information. [file 41598_2024_64054_MOESM1_ESM.docx]

**Supplementary Materials**

**Cortical activities of multisensory contextual plausibility modulation of vibrotactile perception in virtual environments in middle-aged and old adults**

Kathleen Y. L. Kang^1,2*^, Robert Rosenkranz^1,3^, Ercan Altinsoy^1,3^ and Shu-Chen Li^1,2*^

^1^Centre for Tactile Internet with Human-in-the-Loop (CeTI), Technische Universität Dresden,

Dresden, Germany

^2^Chair of Lifespan Developmental Neuroscience, Faculty of Psychology, Technische

Universität Dresden, Dresden, Germany

^3^Chair of Acoustics and Haptics, Faculty of Electrical and Computer Engineering, Econiche

Universität Dresden, Dresden, Germany

Corresponding authors: Shu-Chen Li ([shu-chen.li@tu-dresden.de](mailto:shu-chen.li@tu-dresden.de)) and Kathleen Y. L. Kang ([kathleen_yen_li.kang@tu-dresden.de](mailto:kathleen_yen_li.kang@tu-dresden.de))

***Comparing congruence-based plausibility modulation of cortical activity observed in older ages and in young adulthood***

To directly compare the current data obtained from middled-aged and old adults with previous effects observed in young adults, we conducted secondary analyses combining the dataset from our previous study of young adults (Kang et al., 2022) with the data collected from the current sample. Potential age-related difference in experiences with computer/video games was tested with respect to a self-report measure of usage frequency on a 6-point scale (see Methods for details) using the Mann-Whitney U Test. No significant difference was observed (W=720.5, p=0.41) between young adults (Median =1, IQR= 1-2) and the current sample middled-aged and old adults (Median = 1, IQR=1-2).

In this secondary analysis, data from the previously published young adults data were adjusted with age-dependent partial pathlength factor (PPF) based on the mean age of young adults (mean age=23.86, DPF_760nm_=6.12, PPF_760nm_ =0.10, DPF_850nm_=5.06, PPF_850nm_=0.08), while the data of middle-aged and old adults were adjusted with age-dependent PPF based on their mean age (mean age=62.36, DPF_760nm_=7.17, PPF_760nm_ =0.12, DPF_850nm_=6.10, PPF_850nm_=0.10) to allow for a direct age-related comparison (cf. Scholkmann and Wolf, 2013; . Whiteman et al., 2018) on how congruence-based plausibility affects cortical activity in virtual-reality scenarios.

Based on activities across all channels, overall there was a significant main effect of ‘Plausibility’, *F*(1, 7167)=96.74, *p*<0.0001, *η_p_^2^*=0.01, while the main effects of ‘Scene’, *F*(1, 7167)=0.68, *p*=0.41, *η_p_^2^*=0.00009 or ‘Age’, *F*(1, 67)=0.31, *p*=0.58, *η_p_^2^*=0.0047 were not significant. However, there were significant 2-way and 3-way interactions involving age: Plausibility x Age interaction, *F*(1, 7167)=23.03, *p*<0.0001, *η_p_^2^*=0.003, Scene x Age interaction, *F*(1, 7167) = 10.35, *p*=0.0013, *η_p_^2^*=0.003, and Scene x Plausibility x Age interaction, *F*(1, 7167)=4.29, *p*=0.038, *η_p_^2^*=0.0006 (see **Fig. S1**). The three-way interaction was driven by a greater hemodynamic response in younger adults under high plausibility condition for both cobblestone and highway as compared to low plausibility condition (cobblestone: *t*(7167)= 5.81, *Adjusted p*<0.0001, *d*=0.22, *CI*=[0.0000007 0.0000023]; highway: *t*(7167)=8.90, *Adjusted p*<0.0001, *d*=0.33, *CI*=[0.0000015 0.0000031]). The same effects in older adults were marginal (cobblestone: *t*(7167)= 2.94, *Adjusted p*=0.09, *d*=0.11, *CI*=[-0.000000049 0.0000016]) and greatly attenuated (highway: *t*(7167)=1.87, *Adjusted p*>0.99, *d*=0.07, *CI*=[-0.00000034 0.0000013]). A separate control analysis also included the self-report measure of “computer/video gaming experiences” into the analysis of age comparisons as a covariate. Albeit individual differences in this measure being significant, F(1,64)=5.67, p=0.02, *η_p_^2^*=0.08), none of the above reported significant main effect and interaction effects with age were affected (*ps* remained < 0.004 *for main effect and 2-way interactions* and < 0.025 for the 3-way interaction).


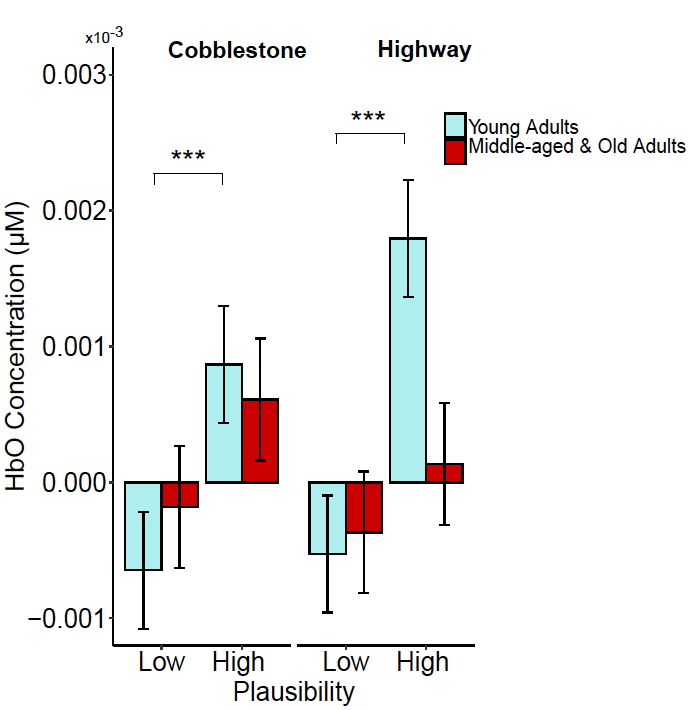


**Fig S1. Estimated marginal means of HbO concentration levels across all channels for low and high plausibility scenarios for the extreme rough (cobblestone) and smooth (high) scenes in young as well as middle-aged and old adults (results based on age-adjusted pathlength).** Error bars represent ± 1 standard error of mean.

***Results based on deoxygenated haemoglobin (HbR)***

We also used linear mixed-effects models to analyze HbR data of old adult participants (*N*=33) that were collected in the current study, with road scene and plausibility as fixed-effects while participants with NIRS channels nested in participants as random intercepts, to investigate the effect of congruence-based plausibility of vibrotactile stimulation on HbR concentration levels when participants passively viewed audio-visual car-riding scenes and received congruent or incongruent vibrotactile stimulations. The main effects of ‘Scene’, *F*(3, 8106)=4.65, *p*=0.003, *η_p_^2^*=0.0017 and ‘Plausibility’, *F*(1, 8106)=19.27, *p*<0.0001, *η_p_^2^*=0.0024 were significant; but the ‘Scene’ x ‘Plausibility’ interaction was not significant, *F*(3, 8106)=1.51, *p*=0.21, *η_p_^2^*=0.00056 (see Fig. S2),


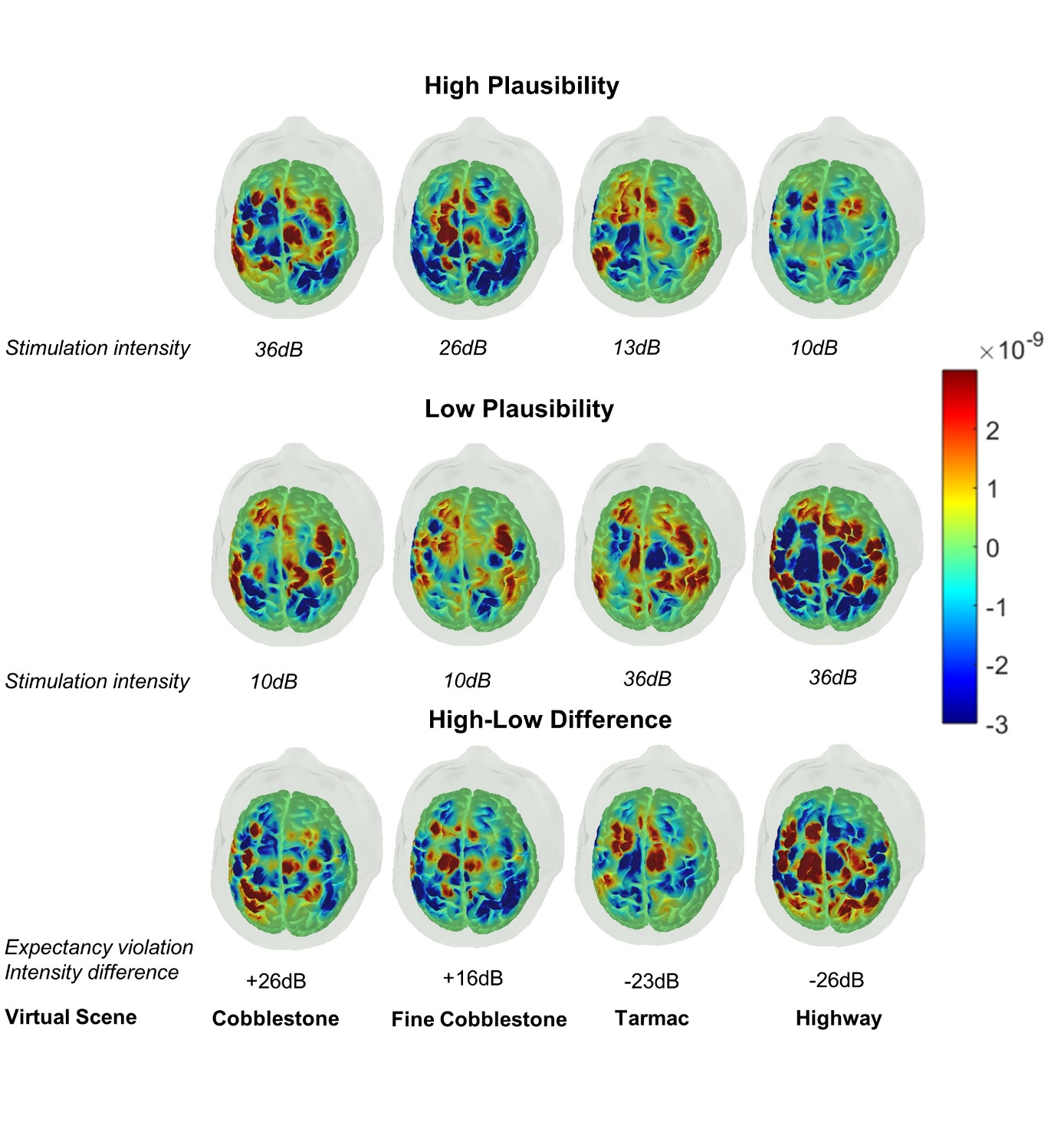


**Fig. S2** **Topographical plots of levels of HbR concentration across all channels. The different columns correspond to the four different road scenes**. The top and middle rows show HbR concentration from high and low plausibility scenarios respectively, whereas the third row depicts the difference plots (high-low plausibility).

**References**

Kang, K., Rosenkranz, R., Karan, K., Altinsoy, E., & Li, S. C. Congruence-based contextual plausibility modulates cortical activity during vibrotactile perception in virtual multisensory environments. *Commun. Biol.* **5**, 1360, 1-13 (2022).

Scholkmann, F., & Wolf, M. (2013). General equation for the differential pathlength factor of the frontal human head depending on wavelength and age. *J. Biomed. Opt.* **18**, 105004. (2013).

Whiteman, A. C., Santosa, H., Chen, D. F., Perlman, S., & Huppert, T. Investigation of the sensitivity of functional near-infrared spectroscopy brain imaging to anatomical variations in 5-to 11-year-old children. *Neurophotonics*, **5**, 01100 (2018).
